# Pharmacological or non-pharmacological interventions for treatment of common mental disorders associated with Tuberculosis: A systematic review

**DOI:** 10.1177/14799731211003937

**Published:** 2021-04-26

**Authors:** Saeed Farooq, Jessica Tunmore, Rifat Comber

**Affiliations:** 1 School of Medicine, 4212 Keele University, Staffordshire, UK; 2 7586 Midlands Partnership Foundation Trust, St George’s Hospital, Stafford, UK

**Keywords:** Common mental disorders, Tuberculosis, interventions, depressive disorders, psychosocial interventions

## Abstract

We aimed to review the literature on interventions for treating Common Mental Disorders (CMD) in people with Tuberculosis (TB). We followed PRISMA guidelines and the protocol was registered at PROSPERO. The electronic databases (PsycInfo, CINAHL, Medline, Google Scholar, Embase) were searched from 1982 to 2020. 349 relevant records were screened, with 26 examined at full text. 13 studies were included totalling 4326 participants. A meta-analysis was not possible due to nature of data, thus descriptive synthesis was conducted. Eleven studies evaluated psychosocial interventions, which significantly improved adherence or cure rates from TB, anxiety and depression. The elements of effective psychosocial interventions included; combating stigma, socioeconomic disadvantage, managing associated guilt and fear of contagion, and explanatory models of illness in local population. Two articles evaluated pharmacological interventions (antidepressants and Vitamin D). This is the first systematic review of interventions to treat CMD in TB. The studies were mostly low quality and mental health outcomes were not adequately described. However, this review suggests that it is feasible to develop and test interventions for improving mental health outcomes and enhancing treatment adherence in TB.

## Introduction

Tuberculosis (TB) is one of the top 10 causes of death and the leading cause from a single infectious agent (above HIV/AIDS).^1^ Common mental disorders (CMD), an umbrella term for depression (and subthreshold disorders) and anxiety disorders, are major multimorbidities associated with TB.^2,3^ Common Mental Disorders include the following psychiatric disorders: depression, generalised anxiety disorder (GAD), panic disorder, phobias, social anxiety disorder, obsessive-compulsive disorder (OCD) and post-traumatic stress disorder (PTSD).^4^

Depression is a common condition among the CMD studied in relation to TB and Multi Drug Resistant TB (MDR-TB).^5^ The prevalence of depression in patients receiving treatment for TB ranges from 11.3% to 80.2%, with a mean weighted prevalence of 48.9%.^5^ A recent scoping review found varying prevalence rates of anxiety amongst Tuberculosis patients, ranging from 2% to 27%.^6^

The relationship between depression and TB is bidirectional. Depression is associated with a range of adverse outcomes in TB such as poor functional impairment, poor adherence to medication and self-care regimens, increased medical symptom burden and increased mortality.^7,8^ Depression may also increase risk of TB reactivation, antibiotic drug resistance, contribute to disease progression, and/or inhibit the physiological response to anti-tuberculosis treatment (ATT). Conversely, TB may precipitate depression, as a result of altered inflammatory responses and/or dysregulation of the hypothalamic-pituitary-adrenal axis, or the side effects of ATT.^9^ This results in worse prognoses for TB, treatment with drugs that have significant neuropsychiatric side effects, enhanced stigma and social isolation. These factors then lead to further depression and anxiety.^9,10^

Common mental disorders, especially depression may be linked to non-adherence to ATT, which is a major barrier in Tuberculosis control.^11^ Untreated depression at baseline is independently associated with greater disability, poorer quality of life,^12,13^ TB treatment default^12^ and negative TB treatment outcomes.^14^ Poor adherence with TB treatment leads to multi drug resistant TB (MDR-TB)^5^ MDR-TB is declared as a health security threat by WHO.^1^

In view of the high prevalence of CMD in TB and their deleterious effects on TB outcomes, the interventions to treat CMD in TB will have major public health advantages. At present the interventions to treat CMD in association with TB have not been systematically reviewed.

We therefore aimed to review the literature on interventions used for treating CMD in people with TB. We included both pharmacological and non-pharmacological interventions. The non-pharmacological interventions include any psychosocial interventions that are focused on psychological or social factors, including, but not limited to, individual, family, or group psychological therapies, education, or training.^15^ We will refer to non-pharmacological interventions as psychosocial interventions in the rest of article.

Our primary aim was to identify the interventions for treating CMD and how these can be implemented in TB control programmes. Specifically, we aimed to answer following questions:

- What interventions have been used for treating CMD in people with TB?
- What is the evidence of effectiveness of psychological, pharmacological and psychosocial interventions for treating CMD in TB?
- What is the methodological quality of the evidence that is available for treating CMD, particularly depression and how can this can be scaled up?

## Materials and methods

We followed the Preferred Reporting Items for Systematic Reviews and Meta-Analyses (PRISMA) statement guidelines.^16^ A protocol defining the key methodological parameters was developed prior to the literature search and was registered at the International Prospective Register of Systematic Reviews (PROSPERO) (https://www.crd.york.ac.uk/PROSPERO/display_record.php?RecordID=176124). The protocol was revised (April, 2020 and July 2020). These were to expand the search date parameters, and to include all common mental disorders.

## Search strategy

The following electronic databases and platforms were searched from 1982 to 2020; PsycInfo, CINAHL, Medline, Embase, Pubmed and Google Scholar. Tuberculosis is mostly prevalent in low and middle income countries (LAMI), and therefore the search strategy was adapted to be reflective of this. Most journals from LAMI countries are not indexed in the mainstream databases; therefore, Google Scholar was used to identify relevant studies. We have used this search strategy previously to identify literature from LMIC.^17^ WHO estimates were used to determine the 20 countries where TB prevalence is highest. Each of these countries were added to the existing search strategy, and the first 40 results from Goggle Scholar were examined for relevant articles. We also searched the bibliography of included studies and four relevant reviews on epidemiology of depression in people with TB.^14,18,19^

The following search terms were used: Depression (all variants) mood disorder* (all variants) neurosis (all variants) anxiety (all variants) common mental health problem, Tuberculosis, TB, multi-drug resistant tuberculosis, MDR-TB, anti-tuberculosis treatment, ATT, rifampicin, ethambutol, INH streptomycin isoniazid. The following were used to search for psychosocial interventions; psychological treatment, psychotherapy, cognitive behaviour* therapy (all variants) counselling, behavioural activation (all variants) OR interpersonal therapy OR psychodynamic psychotherapy OR psychosocial treatment*, befriending, non-pharmacological, self-help, counselling, interpersonal psychotherapy. Pharmacological interventions were searched for using pharmacological treatment* OR *drug treatment* or *Antidepressants* (All variants). Truncations and related terms were used as appropriate based on individual database procedures (please see Online Appendix for search strategy).

We included all studies that reported interventions for CMD. For the purpose of this review we adopted study defined criteria for diagnosis of CMD. Furthermore, the study defined diagnosis of TB or MDR-TB was used. Interventions for MDR-TB were also included. Studies describing patients above 5 years old were included.

We employed a broad definition of psychosocial interventions. This included studies that reported any interventions with psychological or psychosocial elements addressing the psychological consequences of suffering from TB using a broad approach. These approaches could be based on a defined psychological approach such as Cognitive Behavioural Therapy (CBT) (a talking therapy that helps you manage difficulties by changing the way you think), counselling, interpersonal therapy (therapy focusing on your relationships with people) and behavioural activation (increasing pleasurable activity) or any combination of these. Pharmacological interventions included any pharmacological interventions used for treating anxiety or depression. Articles that aimed to prevent rather than treat anxiety and depression reported in TB due to Bovine TB were excluded.

### Data extraction and collection

After completing the search process and screening for relevant abstracts, examination of full text articles for inclusion and data extraction was completed by two reviewers (SF & RC) independently, as outlined in the protocol.^20^ Data extraction began on 14/04/2020. Disagreements regarding study selection or data extraction were resolved by consensus or by a third reviewer (JT) where needed. References of articles were also manually searched to identify additional relevant articles.

A data extraction sheet was developed based on the pre-specified outcomes. Relevant data was extracted by one reviewer (JT) and cross checked by another reviewer (RC). Studies were categorised in terms of; objectives of the study, methodology, (quantitative or qualitative), design, setting, key findings and interventions (pharmacological and non-pharmacological).

### Risk of bias (quality assessment)

The quality of the studies was assessed independently by two reviewers using appropriate checklists. The intervention studies were assessed using Cochrane collaboration tool and for observational studies and we planned to use the Newcastle-Ottawa tool for non-intervention studies. The quality of intervention studies using RCT design was assessed based on the seven principles of risk of bias assessment tool and were categorised as having; low, high and unclear risk of bias for each item in the tool.

## Results

The electronic searches returned 349 relevant abstracts and titles. These included 186 records from electronic searches of the scientific literature databases (PubMed, Psychinfo, Medline & CINHAL) and 200 from the Google Scholar search. We screened the titles and abstracts and excluded the studies which were not directly relevant to the objectives of the review. After screening these titles and abstracts and removal of duplications we decided to examine 26 full text papers. Finally, we included 13 papers in the review.

**Figure 1.**
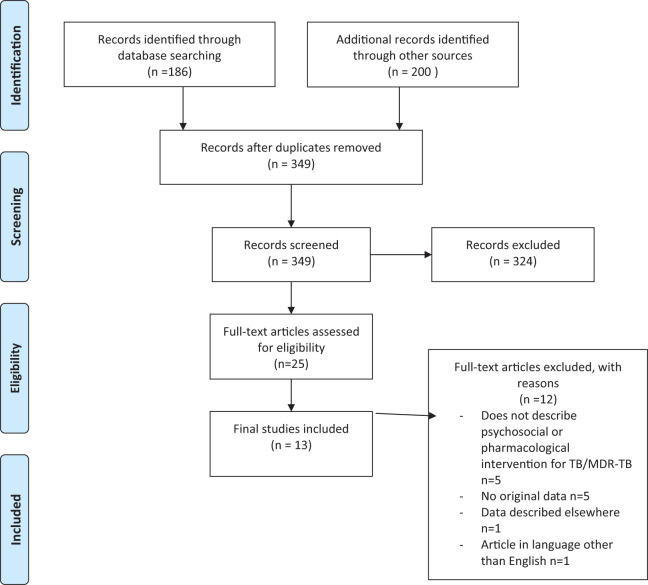
PRISMA diagram.

Out of the total included studies, four^21–24^ were identified through Google search and the remaining were identified through the scientific databases. The details of search yield and reasons for excluding the full text articles are provided in Figure 1.

Figure 1 denotes the PRISMA diagram which shows search process that was carried out in selecting texts for final review. 12 articles were excluded at full text phase, with reasons for exclusions are given, leaving 13 studies for final inclusion.

### Characteristics of included studies

Thirteen studies met the inclusion criteria; 2 used pharmacological interventions and 11 of these studies focussed on different psychosocial interventions. Eleven studies were published from LMIC countries; Peru (3), India (2), Nepal (2), East Kazakhstan (1), Pakistan (1), Indonesia (1) and Ethiopia (1). Two studies were published from China which is now classified as upper middle income country.

Total numbers of participants in all studies were 4326. This comprised of TB (n = 1005) and MDR-TB (n = 1572) patients. The studies also include 1742 participants who were household contacts with no TB diagnosis^25^ and nurses (n = 7).^22^

Eleven studies described psychosocial interventions. These included pharmacists led patient education intervention^26^ psychological counselling and health education^27,28^ behaviour modification using psychotherapy,^29^ and psychological therapy to treat depression.^28^ Li and colleagues^30^ describe a multifaceted intervention including health education, psychotherapy and psychoeducational workshops. Four studies described psychosocial interventions for MDR-TB.^21,23–25^ One study describes the emotional support given by nurses to MDR-TB patients.^22^ These studies used widely different methodologies from qualitative studies describing developing the intervention development, process to RCTs evaluating these interventions. Two studies evaluated pharmacological interventions.^31,32^

Table 1 describes the characteristics of the final included studies in this review, including; author, year, country, setting, population, method, design and sample characteristics.

**Table 1.** Characteristics of included studies.

Author/Year	Population/setting	Country	Method/Design	Sample characteristics and the size
Kaukab et al., 2015	MDR-TB patients in hospital	Pakistan	Mixed Methods (Qualitative and Quantative)	Intervention N = 35
			control trial	Control = 35
Tola et al., 2016	TB Patients across 30 Health Centres in community	Ethiopia	Quantative Randomised Control Trial	Intervention N = 368
				Control N = 330
Janmeja et al., 2005	Pulmonary extrapulmonary TB in National Tuberculosis Programme	India	Quantitative Prospective Control Trial	Intervention N = 100
				Control N = 100
Zhang et al., 2018	Recurrent TBD patients with depression diagnosis	China	Pilot double-blind RCT	Intervention N = 56
				Control N = 64
Kaliakbarova et al., 2013	MDR-TB patients starting second-line drug MDR-TB treatment in community outpatients	Kazakhstan	Qualitative	N = 228
Chalco et al., 2006	MDR-TB receiving nurse led intervention community and health care centres	Peru	Qualitative	N = 7
Meghnani et al., 1988	Pulmonary TB, hospitalised patients	India	Randomised Control Trial	Patients screened but no depression: 51
				Intervention N = 30
				Control N = 29
Acha et al., 2007	MDR-TB Patients	Peru	Qualitative case report	Intervention N = 285
Rocha et al., 2011	MDR-TB patients and their household	Peru	Mixed method (Qualitative and Quantative) operational assessment study	N = 2078 (of which 366 had TB)
Walker et al., 2018	Patients from National	Nepal	Feasibility study, mixed methods (Quantative and Qualitative)	Intervention N = 135
	MDR-TB Programme			
Baral et al., 2014	MDR-TB Patients across seven DOTS centres	Nepal	Mixed methods (Qualitative and Quantative) Pilot intervention study	Intervention N = 75
				Control N = 81
Sari et al., 2020	Pulmonary TB	Indonesia	Quantative quasi experimental	Intervention N = 28
				Control N = 28
Li et al., 2019	Community dwelling Elderly (65 years +) patients with TB	China	Quantative randomised control community trail	Intervention = 61
				Control = 122

### Characteristics of interventions

Different psychosocial interventions were used for improving treatment adherence with ATT and mental health outcomes. Three studies^23,24,26^ used group therapy approaches, four studies^22,29,28,33^ used individual therapy and four papers^21,25,27–30^ described both individual and group approach in providing psychosocial interventions. The number of individual and group sessions differed widely between studies but mostly consisted of 8–10 sessions.

The interventions were provided by nurses,^22,24,25,28^ a psychiatrist and/or nurses,^23^ pharmacists,^26^ lay health workers,^27^ trained health professionals,^33^ combination of health workers and therapists^30^ and psychologists.^21,29^

Five studies provided a theoretical orientation of the intervention used in treatment. Walker and colleagues^27^ used a Healthy Activity Program (HAP) approach which is based on behavioural activation (BA) therapy. Tola et al.^33^ described a therapy based on health belief models of the local population about anxiety and depression. Sari et al.^28^ used Acceptance and Commitment Therapy (ACT),^34^ which was also based on the Health Belief Model. ACT focuses on accepting difficult feelings and emotions to enhance psychological flexibility.^34^ Janmeja and colleagues^29^ used a psychological intervention based on behaviour therapy and Motivational Enhancement Therapy (MET) of Prochaska and DiClemenete.^35^ Li et al.^30^ used Morita therapy,^36^ a kind of cognitive behavioural therapy, which focused on shifting the attention away from the disease to their daily life and external environment.

Two pharmacological intervention studies used Imipramine 75 mg^32^ and Vitamin D supplementation^31^ for treatment of depressive symptoms in people with TB.

Table 2 describes and summarises the characteristics of the psychosocial interventions that were used to treat psychological problems in people with TB and MDR-TB. Individual therapy, group therapy and a combination of the two approaches are described here.

**Table 2.** Characteristics of psychosocial interventions used in people with TB and MDR-TB here.

Study	Intervention Characteristics			
	Frequency	Duration of therapy and sessions	Format	Content
Acha et al., 2007	Weekly at first and then bi-monthly	Eight groups that ran for	Group therapy with 8–12 patients in each group	Psychosocial support, recreational excursions, symbolic celebrations, family workshops, instilling hope to fight stigma
		5 years		
		Number of sessions not specified		
Bara et al., 2014	Counselling was provided every 2–3 weeks.	Not specified	Group therapy	Counselling combined with financial support to help address stigmatisation
Kaukab et al., 2015	Counselling given monthly, also monthly food basket and free medicine	Ten months	Group sessions	Socio-economic help, health education, counselling focussing on personal relationships and problems in the work place
		Number of sessions not specified		
Tola et al., 2016	Health education interventions and counselling sessions provided once a week in the first month, and then monthly	Health education sessions across 4 months	Individual sessions	Patient education based on six Health Belief Model domains including TB disease acquisition, risks of non-adherence to treatment, overcoming psychological barriers, developing self-efficacy
		Sessions lasted 30mins		
		Seven sessions		
Janmeja et al., 2005	Fortnightly sessions during the first 2 months of treatment, and then at monthly intervals	Six months	Individual sessions	Psychotherapy based on behaviour therapy and Motivational Enhancement Therapy (MET)
		Eight sessions of psychotherapy		
		Initial sessions		
		1hour long, subsequent sessions 30–45 mins		
Chalco et al., 2006	None specified	Emotional support that nurses provided across a patients care (various lengths).	Individual sessions	Emotional support and befriending for treatment adherence and addressing stigma, guilt, side effects and socioeconomic difficulties.
Sari et al., 2020	Weekly	Four weeks	Individual sessions	Acceptance and commitment therapy based on; health beliefs about the disease, patient’s values, and the self in context and commitment to action and treatment adherence
		Four sessions lasting 30–40 mins		
Walker et al., 2018	Counselling: not specified	Duration not specified	Individual and group sessions	Stepped care model including educational materials, group support, screening for depression & individual counselling based on the Healthy Activity Program (HAP) approach to address depression and issues of low social support
	‘Regular’ group meetings	Counselling: eight sessions lasting 30–60 mins each		
		8–15 patients met for group support for up to an hour		
Rocha et al., 2011	Not specified	Communities had been receiving socioeconomic interventions for between 3 and 34 months and the time of analysis	Individual and group sessions	Socio-economic intervention focusing on TB patients and their household contacts Home visits to all TB affected households, fortnightly community mobilisation workshops, income generation, psychological counselling, and microenterprise
		Number of sessions not specified		
Kaliakbarova et al., 2013	Not specified	Nine months	Individual & group sessions	Psychosocial support, food and hygiene parcels, home visits, counselling and assistance with finances
		Number of sessions not specified		
Li et al., 2019	Twice a month	Duration not specified	Individual and group sessions	Health education, home visits to support patients in their daily living, peer support, psychotherapy
		Health education lasted 30 mins		
		Psychotherapy – Morita therapy sessions lasted 30 mins		

### The treatment outcomes

We planned a meta-analysis of mental health outcomes for CMD. The studies were conducted in number of different populations, settings and used different interventions and diverse methodologies. Due to the diverse methodologies, study designs and inconsistent, and in some cases poor, reporting of mental health outcomes, it was inappropriate to pool the data to produce a statistical summary. We therefore describe the main findings and produce a narrative summary of results.

Kaukab et al.^26^ used Becks Depression Inventory to measure the outcome of a pharmacy-based intervention. Depression improved significantly at 10 months’ post intervention compared to baseline (moderate depression baseline 54.26% versus 11.43% post intervention, and severe depression 37.14% at baseline versus zero at the end of intervention).

In a prospective controlled trial, Janmeja et al.^29^ evaluated the role psychotherapy (eight sessions) versus treatment as usual in India. Hamilton Rating Scale for depression (HSRD) was used pre-intervention. Treatment adherence and cure rates for TB improved in the intervention group compared to the control; (83% versus 47% and 72% versus 42% respectively). The psychotherapy resulted in improvement in measures such as motivation to take drugs and disease related anxiety but no specific improvement in depression was reported in the study

A cluster randomised control by Tola et al.^33^ evaluated the effectiveness of a psychological intervention that had two components; anxiety and depression counselling and patient education. The study reported a significant improvement in treatment adherence (treatment non-adherence in the intervention group [9.5%] versus 25.4% in control group; p < 0.001). Mental health outcomes were measured at baseline using Kessler–10 (K-10) item questionnaire. The intervention resulted in significant improvement in psychological distress (mean score on Kessler-10 item scale 14 [SD ±5] in the intervention group versus 19 [±8.1] among control group at the end of follow up period). The intervention groups also had significant improvement in the knowledge about the disease, perceived susceptibility, and perceived self-efficacy.

Four sessions of Acceptance and Commitment Therapy (ACT), based on the Health Belief Model was used by Sari et al.^28^ ACT resulted in significant improvement in depression as measured by Beck Depression Inventory (BDI) (mean BDI in intervention group 5.96 SD 1.45 compared to the mean BDI of 7.86 SD 3.05 in control group; P > .001).^28^

Walker et al.^27^ described a feasibility study of a stepped care approach for treatment of anxiety and depression in TB. Patients were screened for depression and anxiety (Hopkins Symptom Checklist, HSCL) and low social support (using the Multidimensional Scale of Perceived Support, MSPSS) monthly in TB control programme. Those who screened positive on either tool (anxiety ≥17, depression ≥24 on HSCL or ≤3 on MSPSS) received Health Activity Programme (HAP) intervention (brief counselling based on behavioural activation theory). In total, 29 participants were referred for counselling. The patients who completed HAP counselling had a mean reduction in scores on both depression tools (7.9 points on HSCL Depression, 2.7 points on PHQ-9).

Baral et al.^24^ used mixed methods, comprising a formative qualitative study and a pilot intervention. The pilot intervention study compared three groups; ‘counselling’, ‘combined counselling and financial support’ and ‘care as usual’. Qualitative interviews revealed participants felt their negative thoughts decreased, and self-esteem increased. Counselling alone and combined counselling and financial support were valued by patients. The following cure rates from TB were found; supported group counselling (85%), support group counselling & financial support (76%) and no support (54%). No specific mental health outcomes were reported by authors; however participants in both supported groups reported improved understanding of TB and were able to solve many problems related to its treatment.

Kaliakbarova et al.^21^ reported a qualitative pilot study of a psychosocial support programme (PSSG) intervention. PSSG was implemented for 226 patients considered to be of high risk of MDR-TB treatment default. They found that prior to the start of the programme, 44 (23%) of 190 patients had interrupted TB treatment at least once. After the PSSG, only one patient (0.5%) had interrupted treatment only once. Half of participants in the study (n = 95) considered that the psychological support provided by the programme helped them to recover from the disease.

Rocha et al.^25^ evaluated a ‘socioeconomic support intervention’ in a mixed method study. Baseline depression scores were measured using the Beck Depression Inventory. Patients and household contacts had high rates of depression at baseline; 40% of TB patients had any depression and 12% had severe depression. They found that the intervention resulted in successful TB treatment completion, better testing for HIV (increase from 31% to 97%) and completion of preventative therapy (from 27% to 87%). Depression scores were not given post intervention.

Chalco et al.^22^ explored the forms and means of emotional support that nurses could provide to patients living with MDR-TB in Peru using ethnographic and qualitative methods. Theme and content analyses of data revealed 10 issues related to MDR-TB care mostly related to implementing psychosocial interventions (see below). The study did not report specific mental health outcomes.

Acha et al.^23^ found that the psychosocial groups led by teams of psychiatrists and nurses helped patients with MDR-TB to cope with TB associated stigma as they experienced social rejection and discrimination from family members. The study did not report specific mental health outcomes but provides qualitative data from the support groups, on therapeutic processes to overcome depression and suicidal tendencies. One powerful therapeutic effect of the group was the inspiration of hope in participants including a sense of belonging and cohesion. Suicide featured frequently in discussions such as

All of us go through that (suicidal ideation); that’s normal. The beginning of treatment is so hard, but it gets easier. I thought about killing myself many times. The side effects were so bad; – But little by little, things got better. We have to be strong. We can’t give up. The most important thing to know is that you’re not alone. We’re all in this together.^23^

Li et al.^30^ used the Zung self-rating Anxiety Scale (SAS) and the Zung self-rating Depression Scale to measure the outcome of a psychosocial intervention to improve anxiety and depression among elderly TB patients. This was the only study that looked specifically at the elderly TB population. At 6 months follow up, significant improvement was found both for anxiety and depression in the intervention group compared to the control group.

### Pharmacological treatments

Meghnani et al.^32^ compared Imipramine (25 mg) versus placebo. Significantly higher number of patients improved on imipramine versus control group (86.7% versus 48.3% in the control group; P < 0.01) using a cut-off point of 13 on HDRS as criteria for remission from depression.

In a pilot RCT, Zhang et al.^31^ used vitamin D in 120 patients (56 in vitamin D group versus 64 control) presenting with pulmonary TB and meeting the DSM-IV criteria for depression. Change in scores of depressive symptoms was measured with the Chinese version of the Beck Depression Inventory (BDI). After 8-week treatment, although the BDI scores in both the control and intervention decreased significantly from baseline, the mean BDI scores were not significantly different between intervention (16.6 ± 9.4) and the control group (16.9 ± 8.3); P = 0.38.

### Quality assessment of included studies

**Figure 2.**
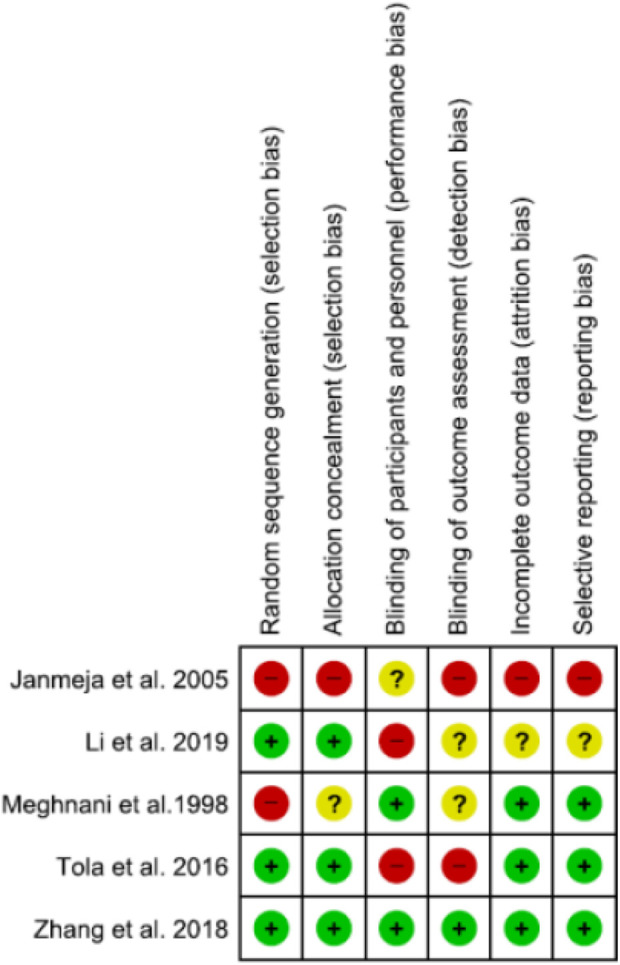
Risk of bias assessment.

Seven studies used experimental designs to evaluate the interventions but most provided little details on the quality criteria for Cochrane tool for assessment of RCTs. One study^26^ was described by authors as RCT and another was described as quasi experimental.^28^ Both studies did not provide any details relevant to quality criteria for RCT, so the Cochrane tool could not be applied to these studies. The quality assessment of remaining five studies is provided in Figure 2. Only one study^31^ had a low risk of bias score on all the items on Cochrane tool for the assessment of intervention studies, the rest were classified as having high risk of bias.

Figure 2 denotes the results of the quality assessment that was carried out by the reviewers. This figure demonstrates that only one figure scored as low risk of bias, with the other studies scoring high.

Six studies used observational designs and were suitable for quality assessment using the tool proposed in the protocol. Two studies^22,23^ did not use appropriate study designs, and did not provide relevant information for using the Newcastle-Ottawa Quality Assessment Scale (NOQAS) that we proposed in the protocol for observational studies.

The NOQAS provides 1–3 stars for each item such as such as description of cohorts, outcome and ascertainment of exposure, and we converted this to a numerical score. The maximum score for a study is 10. Out of six studies, four studies used a validated measurement tool to measure mental health outcomes.^25–28^ One study (Sari et al.)^28^ scored 7 out of a possible 10 stars, two studies scored 6,^26,27^ one study scored 5^25^ one study scored 4^24^ and one study^21^ scored 2 out of a possible 10.

## Discussion

This is the first systematic review of interventions to treat common mental disorders in people with TB and MDR-TB. We used a broad inclusion criterion to examine all possible designs and interventions to inform research and clinical practice. Since a meta-analysis was not possible, it is not possible to provide quantitative outcomes for the interventions. The main findings are (i) All studies reported improvement in adherence or cure rates from TB/MDR-TB, either in comparison to a control group or in comparison to pre-intervention rates with the use psychosocial interventions, (ii) specific outcomes related to anxiety and depression were not adequately described but most studies reported improvement in mental health outcomes, (iii) studies provided rich information about the components of psychosocial interventions, (iv) Importantly, these studies showed that psychosocial interventions can address both mental health problems and adherence with Anti-Tuberculosis treatment (ATT), the two most significant barriers in TB control worldwide.

In view of the fact that meta-analysis could not be done, it is not possible to provide quantitative estimates of any intervention, however the narrative review of the studies provides useful insights into the development of interventions and elements of interventions that can be used in future scale up. The psychosocial interventions addressing CMD need to focus on stigma, socioeconomic disadvantage, guilt of making family members vulnerable, fear of contagion, health belief models, developing self-efficacy and support for family members. Therapies will need a broad transdiagnostic focus, and therapeutic approaches such as Cognitive Behavioural Therapy and Acceptance and Commitment Therapy could provide theoretical underpinning for future interventions. The evidence suggests that task shifting involving TB health workers, using a transdiagnostic and stepped care approach can be helpful in implementing psychological intervention programme in TB control programmes, in line with literature on scaling up psychosocial interventions in LMIC.^37,38^ A stepped model consisting of detecting CMD using a simple screening questionnaire, and providing care for those with emotional problems,^27^ appeared to be an effective approach that could be used for scaling up interventions.

Pharmacological treatment of TB in depression is particularly challenging in view of significant interactions between ATT and antidepressants.^39^ Potential interactions include therapeutic failure or toxicity of ATT and/or psychoactive agents and concerns have been raised over the potential for drug interactions between various SSRIs and isoniazid.^39^ Therefore, it is important to examine the efficacy and safety of antidepressants in this population, however only one trial evaluated effect of an antidepressant. One good quality study did not show effectiveness of Vitamin D in TB and depression. Using pharmacological agents such as Vitamin D in TB may be useful strategy for treating depression in this population, as this would avoid possible interaction. Other agents with a potential antidepressant effect such as fatty acids or anti-inflammatory drugs^40^ may potentially be useful in this population and need to be evaluated in future studies.

This study reflects the neglect of research needs of problems facing the LMIC population. In 2018, the 30 high TB burden countries were mostly LMIC and these accounted for 87% of new TB cases worldwide. The significant association between depression and TB and its deleterious effect on TB control has been highlighted by number of studies.^7,9^ TB and depression comorbidity is now described as ‘TB-depression syndemic’^7^ which poses significant risk to the Global End TB Strategy.^9^ Only 13 studies describing both pharmacological and psychosocial interventions to tackle this huge public health problem is a sad reflection of the priorities for research on public health problems faced by LMIC.

### Strengths and limitations

We used a robust methodology following PRISMA guidelines and a comprehensive search strategy that captured all relevant studies on the subject. Only one potentially eligible study could not be included as the full text was in Chinese language.^41^ The information available in the abstract (quasi experimental with 74 participants) of the study showed that this study would not materially change the findings of our review.

The major limitations of this review are that included studies were mostly of low quality, had poorly reported mental health outcomes. We only included English language studies and may have missed studies published in other languages. Publication bias arising from the fact that studies with negative results are less likely to be published may also have affected results.

Almost all controlled trials had high or unclear risk of bias. The observational studies lacked proper control groups and adequate description of participants and outcomes. Despite these limitations, we believe we have identified major components of potentially successful interventions and diagnostic and screening measures that can help to design future studies. We also identify major barriers in implementing these interventions in LMIC that can help in scaling up the interventions for CMD in TB.

## Supplemental material

Supplemental Material, sj-pdf-1-crd-10.1177_14799731211003937 - Pharmacological or non-pharmacological interventions for treatment of common mental disorders associated with Tuberculosis: A systematic review Click here for additional data file. Supplemental Material, sj-pdf-1-crd-10.1177_14799731211003937 for Pharmacological or non-pharmacological interventions for treatment of common mental disorders associated with Tuberculosis: A systematic review by Saeed Farooq, Jessica Tunmore and Rifat Comber in Chronic Respiratory Disease
