# Supplementary material for: Pharmacological or non-pharmacological interventions for treatment of common mental disorders associated with Tuberculosis: A systematic review
Source: Chron Respir Dis. 2021 Apr 26;18:14799731211003937. doi: 10.1177/14799731211003937 (PMC8082988; doi:10.1177/14799731211003937)
Supplement: Supplemental Material, sj-pdf-1-crd-10.1177_14799731211003937 - Pharmacological or non-pharmacological interventions for treatment of common mental disorders associated with Tuberculosis: A systematic review [file sj-pdf-1-crd-10.1177_14799731211003937.pdf]

Appendix 1 – Search Details

**Sources searched:**					
PsycInfo	Y	PubMed	Y		
CINAHL	Y	Google Scholar	Y		
Medline	Y				
Embase	Y				

#	Database	Search term	Results
1	EMBASE	(TB).ti,ab	64101
2	EMBASE	(tuberculosis).ti,ab	174202
3	EMBASE	(1 OR 2)	194293
4	EMBASE	exp TUBERCULOSIS/	184454
5	EMBASE	(3 OR 4)	244408
6	EMBASE	exp "ANTIDEPRESSANT AGENT"/	414612
7	EMBASE	(5 AND 6)	1085
8	EMBASE	(review AND (umbrella OR systematic)).ti,ab	216260
9	EMBASE	("randomi* controlled trial" OR RCT).ti,ab	144126
10	EMBASE	(8 OR 9)	351468
11	EMBASE	(7 AND 10)	26
12	EMBASE	(review* OR trial* OR study OR studies OR research).ti,ab	14334714
13	EMBASE	(7 AND 12)	544
14	EMBASE	(MDR-TB).ti,ab	4926
15	EMBASE	("Multi Drug Resistant" AND (TB OR tuberculosis)).ti,ab	1995
16	EMBASE	("multi-drug resistant" AND (TB OR tuberculosis)).ti,ab	1995
17	EMBASE	("multidrug resistant" AND (TB OR tuberculosis)).ti,ab	7308

18	EMBASE	(14 OR 15 OR 16 OR 17)	10304
19	EMBASE	(6 AND 18)	25
20	Medline	(TB).ti,ab	48916
21	Medline	(tuberculosis).ti,ab	181558
22	Medline	(MDR-TB).ti,ab	3528
23	Medline	("Multi Drug Resistant" AND (TB OR tuberculosis)).ti,ab	1313
24	Medline	("multi-drug resistant" AND (TB OR tuberculosis)).ti,ab	1313
25	Medline	("multidrug resistant" AND (TB OR tuberculosis)).ti,ab	6116
26	Medline	TUBERCULOSIS/ OR "LATENT TUBERCULOSIS"/ OR "PERITONITIS, TUBERCULOUS"/ OR TUBERCULOMA/ OR "TUBERCULOSIS, CARDIOVASCULAR"/ OR "TUBERCULOSIS, CENTRAL NERVOUS SYSTEM"/ OR "TUBERCULOSIS, CUTANEOUS"/ OR "TUBERCULOSIS, ENDOCRINE"/ OR "TUBERCULOSIS, GASTROINTESTINAL"/ OR "TUBERCULOSIS, HEPATIC"/ OR "TUBERCULOSIS, LARYNGEAL"/ OR "TUBERCULOSIS, LYMPH NODE"/ OR "TUBERCULOSIS, MILIARY"/ OR "TUBERCULOSIS, MULTIDRUG-RESISTANT"/ OR "TUBERCULOSIS, OCULAR"/ OR "TUBERCULOSIS, ORAL"/ OR "TUBERCULOSIS, OSTEOARTICULAR"/ OR "TUBERCULOSIS, PLEURAL"/ OR "TUBERCULOSIS, PULMONARY"/ OR "TUBERCULOSIS, SPLENIC"/ OR "TUBERCULOSIS, UROGENITAL"/	176220
27	Medline	(20 OR 21 OR 22 OR 23 OR 24 OR 25 OR 26)	248799
28	Medline	exp "ANTIDEPRESSIVE AGENTS"/	149352
29	Medline	(27 AND 28)	233
30	Medline	(review AND (umbrella OR systematic)).ti,ab	166863
31	Medline	("randomi* controlled trial" OR RCT).ti,ab	102162
32	Medline	(review* OR trial* OR study OR studies OR research).ti,ab	11138219
33	Medline	(30 OR 31 OR 32)	11139293
34	Medline	(29 AND 33)	84
35	PsycINFO	(TB).ti,ab	1165

36	PsycINFO	(tuberculosis).ti,ab	2370
37	PsycINFO	(MDR-TB).ti,ab	42
38	PsycINFO	("Multi Drug Resistant" AND (TB OR tuberculosis)).ti,ab	16
39	PsycINFO	("multi-drug resistant" AND (TB OR tuberculosis)).ti,ab	16
40	PsycINFO	("multidrug resistant" AND (TB OR tuberculosis)).ti,ab	44
41	PsycINFO	exp TUBERCULOSIS/	1172
42	PsycINFO	(35 OR 36 OR 37 OR 38 OR 39 OR 40 OR 41)	2967
43	PsycINFO	exp "ANTIDEPRESSANT DRUGS"/	37778
44	PsycINFO	(42 AND 43)	5
45	PsycINFO	(antidepressant*).ti,ab	37421
46	PsycINFO	(depression).ti,ab	232903
47	PsycINFO	(depression ADJ2 treat*).ti,ab	22101
48	PsycINFO	(42 AND 47)	8
49	EMBASE	(depression ADJ2 treat*).ti,ab	26797
50	EMBASE	(5 AND 49)	37
51	Medline	(depression ADJ2 treat*).ti,ab	25403
52	Medline	(27 AND 51)	21
53	CINAHL	(MDR-TB).ti,ab	615
54	CINAHL	("Multi Drug Resistant" AND (TB OR tuberculosis)).ti,ab	184
55	CINAHL	("multi-drug resistant" AND (TB OR tuberculosis)).ti,ab	184
56	CINAHL	("multidrug resistant" AND (TB OR tuberculosis)).ti,ab	1119
57	CINAHL	(TB OR tuberculosis).ti,ab	21397
58	CINAHL	exp TUBERCULOSIS/	19743

59	CINAHL	(53 OR 54 OR 55 OR 56 OR 57 OR 58)	25921
60	CINAHL	exp "ANTIDEPRESSIVE AGENTS"/	20988
61	CINAHL	(59 AND 60)	15
62	CINAHL	(depression ADJ2 treat*).ti,ab	8981
63	CINAHL	(59 AND 62)	7
64	EMBASE	(TB OR tuberculosis OR MDR-TB).ti,ab	194315
65	EMBASE	("Multi Drug Resistant" AND (TB OR tuberculosis)).ti,ab	1995
66	EMBASE	("multi-drug resistant" AND (TB OR tuberculosis)).ti,ab	1995
67	EMBASE	("multidrug resistant" AND (TB OR tuberculosis)).ti,ab	7308
68	EMBASE	(64 OR 65 OR 66 OR 67)	194315
69	EMBASE	(depression).ti,ab	437019
70	EMBASE	DEPRESSION/ OR "ADOLESCENT DEPRESSION"/ OR "AGITATED DEPRESSION"/ OR "ATYPICAL DEPRESSION"/ OR "CHRONIC DEPRESSION"/ OR "MAJOR DEPRESSION"/ OR "TREATMENT RESISTANT DEPRESSION"/	403325
71	EMBASE	(69 OR 70)	602184
72	EMBASE	(68 AND 71)	1090
73	EMBASE	("psychological therap*" OR psychotherap* OR psychosocial OR "cognitive behavi* therap*" OR "non-pharmacological").ti,ab	205675
74	EMBASE	exp PSYCHOTHERAPY/	242896
75	EMBASE	(73 OR 74)	376769
77	EMBASE	"PSYCHOSOCIAL CARE"/	17968
78	EMBASE	(75 OR 77)	382263
79	EMBASE	(72 AND 78)	62
80	Medline	(TB OR tuberculosis OR MDR-TB).ti,ab	197453
81	Medline	("Multi Drug Resistant" AND (TB OR tuberculosis)).ti,ab	1313

82	Medline	("multi-drug resistant" AND (TB OR tuberculosis)).ti,ab	1313
83	Medline	("multidrug resistant" AND (TB OR tuberculosis)).ti,ab	6116
84	Medline	TUBERCULOSIS/ OR "LATENT TUBERCULOSIS"/ OR "PERITONITIS, TUBERCULOUS"/ OR TUBERCULOMA/ OR "TUBERCULOSIS, CARDIOVASCULAR"/ OR "TUBERCULOSIS, CENTRAL NERVOUS SYSTEM"/ OR "TUBERCULOSIS, CUTANEOUS"/ OR "TUBERCULOSIS, ENDOCRINE"/ OR "TUBERCULOSIS, GASTROINTESTINAL"/ OR "TUBERCULOSIS, HEPATIC"/ OR "TUBERCULOSIS, LARYNGEAL"/ OR "TUBERCULOSIS, LYMPH NODE"/ OR "TUBERCULOSIS, MILIARY"/ OR "TUBERCULOSIS, MULTIDRUG-RESISTANT"/ OR "TUBERCULOSIS, OCULAR"/ OR "TUBERCULOSIS, ORAL"/ OR "TUBERCULOSIS, OSTEOARTICULAR"/ OR "TUBERCULOSIS, PLEURAL"/ OR "TUBERCULOSIS, PULMONARY"/ OR "TUBERCULOSIS, SPLENIC"/ OR "TUBERCULOSIS, UROGENITAL"/	176220
85	Medline	(80 OR 81 OR 82 OR 83 OR 84)	248799
86	Medline	(depression).ti,ab	312744
87	Medline	DEPRESSION/	112135
88	Medline	exp "DEPRESSIVE DISORDER"/	105449
89	Medline	(86 OR 87 OR 88)	379968
90	Medline	("psychological therap*" OR psychotherap* OR psychosocial OR "cognitive behavi* therap*" OR "non-pharmacological").ti,ab	147377
91	Medline	exp PSYCHOTHERAPY/	199358
92	Medline	"PSYCHOSOCIAL SUPPORT SYSTEMS"/	359
93	Medline	(90 OR 91 OR 92)	303986
94	Medline	(89 AND 93)	42203
95	Medline	(85 AND 94)	32
96	PsycINFO	(TB OR tuberculosis OR MDR-TB).ti,ab	2884
97	PsycINFO	("Multi Drug Resistant" AND (TB OR tuberculosis)).ti,ab	16
98	PsycINFO	("multi-drug resistant" AND (TB OR tuberculosis)).ti,ab	16
99	PsycINFO	("multidrug resistant" AND (TB OR tuberculosis)).ti,ab	44

100	PsycINFO	exp TUBERCULOSIS/	1172
101	PsycINFO	(96 OR 97 OR 98 OR 99 OR 100)	2967
102	PsycINFO	(depression).ti,ab	232903
103	PsycINFO	"MAJOR DEPRESSION"/ OR "TREATMENT RESISTANT DEPRESSION"/ OR "ATYPICAL DEPRESSION"/	119738
104	PsycINFO	(102 OR 103)	252063
105	PsycINFO	("psychological therap*" OR psychotherap* OR psychosocial OR "cognitive behavi* therap*" OR "non-pharmacological").ti,ab	199783
106	PsycINFO	exp PSYCHOTHERAPY/	198990
107	PsycINFO	(105 OR 106)	324867
108	PsycINFO	(104 AND 107)	33576
109	PsycINFO	(101 AND 108)	14
110	CINAHL	(TB OR tuberculosis OR MDR-TB).ti,ab	21397
111	CINAHL	("Multi Drug Resistant" AND (TB OR tuberculosis)).ti,ab	184
112	CINAHL	("multi-drug resistant" AND (TB OR tuberculosis)).ti,ab	184
113	CINAHL	("multidrug resistant" AND (TB OR tuberculosis)).ti,ab	1119
114	CINAHL	exp TUBERCULOSIS/	19743
115	CINAHL	(110 OR 111 OR 112 OR 113 OR 114)	25921
116	CINAHL	exp DEPRESSION/	100389
117	CINAHL	(depression).ti,ab	105058
118	CINAHL	(116 OR 117)	142228
119	CINAHL	("psychological therap*" OR psychotherap* OR psychosocial OR "cognitive behavi* therap*" OR "non-pharmacological").ti,ab	66867
120	CINAHL	exp PSYCHOTHERAPY/	169082
121	CINAHL	"SUPPORT, PSYCHOSOCIAL"/	68178
122	CINAHL	(119 OR 120 OR 121)	213592

123	CINAHL	(118 AND 122)	27251
124	CINAHL	(115 AND 123)	13
125	EMBASE	(depression OR "depressive disorder" OR "major depression" OR "major depressive disorder" OR "treatment resistant depression" OR TRD OR "chronic depression" OR "depressive illness" OR dysthymia OR "mood disorder*" OR neurosis OR neuroses OR anxiety OR "common mental health problem*").ti,ab	602374
126	EMBASE	DEPRESSION/ OR "ADOLESCENT DEPRESSION"/ OR "AGITATED DEPRESSION"/ OR "ATYPICAL DEPRESSION"/ OR "CHRONIC DEPRESSION"/ OR "MAJOR DEPRESSION"/ OR "TREATMENT RESISTANT DEPRESSION"/	403325
127	EMBASE	(125 OR 126)	743091
128	EMBASE	("psychological treatment*" OR psychotherapy OR "cognitive behaviour* therapy" OR "cognitive behavior* therapy" OR CBT OR counselling OR "behavioural activation" OR "behavioral activation" OR "interpersonal therapy" OR "psychodynamic psychotherapy" OR "psychosocial treatment*" OR befriending OR non-pharmacological OR self-help OR counseling OR "interpersonal psychotherapy").ti,ab	215960
129	EMBASE	exp PSYCHOTHERAPY/	242896
130	EMBASE	"PSYCHOSOCIAL CARE"/	17968
131	EMBASE	(128 OR 129 OR 130)	404735
132	EMBASE	(127 AND 131)	74635
133	EMBASE	(Tuberculosis OR TB OR "multi-drug resistant tuberculosis" OR MDRTB OR anti-tuberculosis treatment OR "ATT" OR rifampicin OR ethambutol OR INH OR streptomycin OR isoniazid).ti,ab	229302
134	EMBASE	exp TUBERCULOSIS/	184473
135	EMBASE	(133 OR 134)	277626
136	EMBASE	(132 AND 135)	133
137	EMBASE	(pharmacological treatment* OR drug treatment* OR antidepressants OR anti-depressants OR tricyclic OR anxiolytic* OR sedatives OR monoamine-oxidase inhibitor* OR MAOI OR "selective serotonin reuptake inhibitor*" OR SSSRI* OR doxepin OR clomipramine OR amitriptyline OR nortiptyline OR imipramine OR fluvoxamine OR paroxetine OR fluoxetine OR sertraline OR citalopram OR escitalopram OR venlafaxine OR venlafaxine XR OR duloxetine OR trazodone OR bupropion OR mirtazapine OR mianserin OR benzodiazepines OR anti-anxiety OR antianxiety).ti,ab	234839
138	EMBASE	(127 AND 137)	75195

139	EMBASE	(135 AND 138)	150
140	Medline	(depression OR "depressive disorder" OR "major depression" OR "major depressive disorder" OR "treatment resistant depression" OR TRD OR "chronic depression" OR "depressive illness" OR dysthymia OR "mood disorder*" OR neurosis OR neuroses OR anxiety OR "common mental health problem*").ti,ab	434587
141	Medline	DEPRESSION/ OR exp "ANTIDEPRESSIVE AGENTS"/	246837
142	Medline	(140 OR 141)	574435
143	Medline	("psychological treatment*" OR psychotherapy OR "cognitive behaviour* therapy" OR "cognitive behavior* therapy" OR CBT OR counselling OR "behavioural activation" OR "behavioral activation" OR "interpersonal therapy" OR "psychodynamic psychotherapy" OR "psychosocial treatment*" OR befriending OR non-pharmacological OR self-help OR counseling OR "interpersonal psychotherapy").ti,ab	148339
144	Medline	exp PSYCHOTHERAPY/	199358
145	Medline	"PSYCHOSOCIAL SUPPORT SYSTEMS"/	359
146	Medline	(143 OR 144 OR 145)	308124
147	Medline	(142 AND 146)	43551
148	Medline	(Tuberculosis OR TB OR "multi-drug resistant tuberculosis" OR MDRTB OR anti-tuberculosis treatment OR "ATT" OR rifampicin OR ethambutol OR INH OR streptomycin OR isoniazid).ti,ab	236182
149	Medline	TUBERCULOSIS/ OR "LATENT TUBERCULOSIS"/ OR "PERITONITIS, TUBERCULOUS"/ OR TUBERCULOMA/ OR "TUBERCULOSIS, CARDIOVASCULAR"/ OR "TUBERCULOSIS, CENTRAL NERVOUS SYSTEM"/ OR "TUBERCULOSIS, CUTANEOUS"/ OR "TUBERCULOSIS, ENDOCRINE"/ OR "TUBERCULOSIS, GASTROINTESTINAL"/ OR "TUBERCULOSIS, HEPATIC"/ OR "TUBERCULOSIS, LARYNGEAL"/ OR "TUBERCULOSIS, LYMPH NODE"/ OR "TUBERCULOSIS, MILIARY"/ OR "TUBERCULOSIS, MULTIDRUG-RESISTANT"/ OR "TUBERCULOSIS, OCULAR"/ OR "TUBERCULOSIS, ORAL"/ OR "TUBERCULOSIS, OSTEOARTICULAR"/ OR "TUBERCULOSIS, PLEURAL"/ OR "TUBERCULOSIS, PULMONARY"/ OR "TUBERCULOSIS, SPLENIC"/ OR "TUBERCULOSIS, UROGENITAL"/	176220
150	Medline	(148 OR 149)	284352
151	Medline	(147 AND 150)	43
152	Medline	(pharmacological treatment* OR drug treatment* OR antidepressants OR anti-depressants OR tricyclic OR anxiolytic* OR sedatives OR monoamine-oxidase inhibitor* OR MAOI OR "selective serotonin reuptake inhibitor*" OR SSSRI* OR doxepin OR clomipramine OR	541322

		amitriptyline OR nortiptyline OR imipramine OR fluvoxamine OR paroxetine OR fluoxetine OR sertraline OR citalopram OR escitalopram OR venlafaxine OR venlafaxine XR OR duloxetine OR trazodone OR bupropion OR mirtazapine OR mianserin OR benzodiazepines OR anti-anxiety OR antianxiety).ti,ab	
153	Medline	exp "ANTIDEPRESSIVE AGENTS"/	149352
154	Medline	(152 OR 153)	628930
155	Medline	(142 AND 154)	179127
156	Medline	(150 AND 155)	471
157	PsycINFO	(depression OR "depressive disorder" OR "major depression" OR "major depressive disorder" OR "treatment resistant depression" OR TRD OR "chronic depression" OR "depressive illness" OR dysthymia OR "mood disorder*" OR neurosis OR neuroses OR anxiety OR "common mental health problem*").ti,ab	372625
159	PsycINFO	"MAJOR DEPRESSION"/ OR "TREATMENT RESISTANT DEPRESSION"/ OR "ATYPICAL DEPRESSION"/	119738
160	PsycINFO	(157 OR 159)	385182
161	PsycINFO	("psychological treatment*" OR psychotherapy OR "cognitive behaviour* therapy" OR "cognitive behavior* therapy" OR CBT OR counselling OR "behavioural activation" OR "behavioral activation" OR "interpersonal therapy" OR "psychodynamic psychotherapy" OR "psychosocial treatment*" OR befriending OR non-pharmacological OR self-help OR counseling OR "interpersonal psychotherapy").ti,ab	192666
162	PsycINFO	exp PSYCHOTHERAPY/	198990
163	PsycINFO	exp "PSYCHOSOCIAL FACTORS"/ OR exp "PSYCHOSOCIAL DEVELOPMENT"/	165239
164	PsycINFO	(161 OR 162 OR 163)	472339
165	PsycINFO	(160 AND 164)	65408
166	PsycINFO	(TB OR tuberculosis OR MDR-TB).ti,ab	2884
167	PsycINFO	("Multi Drug Resistant" AND (TB OR tuberculosis)).ti,ab	16
168	PsycINFO	("multi-drug resistant" AND (TB OR tuberculosis)).ti,ab	16
169	PsycINFO	("multidrug resistant" AND (TB OR tuberculosis)).ti,ab	44
170	PsycINFO	exp TUBERCULOSIS/	1172
171	PsycINFO	(166 OR 167 OR 168 OR 169 OR 170)	2967

172	PsycINFO	(165 AND 171)	38
173	PsycINFO	(pharmacological treatment* OR drug treatment* OR antidepressants OR anti-depressants OR tricyclic OR anxiolytic* OR sedatives OR monoamine-oxidase inhibitor* OR MAOI OR "selective serotonin reuptake inhibitor*" OR SSSRI* OR doxepin OR clomipramine OR amitriptyline OR nortiptyline OR imipramine OR fluvoxamine OR paroxetine OR fluoxetine OR sertraline OR citalopram OR escitalopram OR venlafaxine OR venlafaxine XR OR duloxetine OR trazodone OR bupropion OR mirtazapine OR mianserin OR benzodiazepines OR anti-anxiety OR antianxiety).ti,ab	120818
174	PsycINFO	exp "ANTIDEPRESSANT DRUGS"/	37778
175	PsycINFO	(173 OR 174)	130539
176	PsycINFO	(160 AND 175)	45160
177	PsycINFO	(171 AND 176)	20
178	CINAHL	exp DEPRESSION/	100476
179	CINAHL	(depression OR "depressive disorder" OR "major depression" OR "major depressive disorder" OR "treatment resistant depression" OR TRD OR "chronic depression" OR "depressive illness" OR dysthymia OR "mood disorder*" OR neurosis OR neuroses OR anxiety OR "common mental health problem*").ti,ab	146797
180	CINAHL	(178 OR 179)	179872
181	CINAHL	("psychological treatment*" OR psychotherapy OR "cognitive behaviour* therapy" OR "cognitive behavior* therapy" OR CBT OR counselling OR "behavioural activation" OR "behavioral activation" OR "interpersonal therapy" OR "psychodynamic psychotherapy" OR "psychosocial treatment*" OR befriending OR non-pharmacological OR self-help OR counseling OR "interpersonal psychotherapy").ti,ab	64733
182	CINAHL	exp PSYCHOTHERAPY/	169228
183	CINAHL	"SUPPORT, PSYCHOSOCIAL"/	68250
184	CINAHL	(181 OR 182 OR 183)	214903
185	CINAHL	(180 AND 184)	29633
186	CINAHL	(MDR-TB).ti,ab	617
187	CINAHL	("Multi Drug Resistant" AND (TB OR tuberculosis)).ti,ab	184
188	CINAHL	("multi-drug resistant" AND (TB OR tuberculosis)).ti,ab	184

189	CINAHL	("multidrug resistant" AND (TB OR tuberculosis)).ti,ab	1122
190	CINAHL	(TB OR tuberculosis).ti,ab	21417
191	CINAHL	exp TUBERCULOSIS/	19756
192	CINAHL	(186 OR 187 OR 188 OR 189 OR 190 OR 191)	25941
193	CINAHL	(185 AND 192)	18
194	CINAHL	(pharmacological treatment* OR drug treatment* OR antidepressants OR anti-depressants OR tricyclic OR anxiolytic* OR sedatives OR monoamine-oxidase inhibitor* OR MAOI OR "selective serotonin reuptake inhibitor*" OR SSSRI* OR doxepin OR clomipramine OR amitriptyline OR nortiptyline OR imipramine OR fluvoxamine OR paroxetine OR fluoxetine OR sertraline OR citalopram OR escitalopram OR venlafaxine OR venlafaxine XR OR duloxetine OR trazodone OR bupropion OR mirtazapine OR mianserin OR benzodiazepines OR anti-anxiety OR antianxiety).ti,ab	113806
195	CINAHL	exp "ANTIDEPRESSIVE AGENTS"/	21004
196	CINAHL	(194 OR 195)	123267
197	CINAHL	(180 AND 196)	20687
198	CINAHL	(192 AND 197)	19
